# *Celastrus aculeatus *Merr. suppresses the induction and progression of autoimmune arthritis by modulating immune response to heat-shock protein 65

**DOI:** 10.1186/ar2268

**Published:** 2007-07-23

**Authors:** Li Tong, Kamal D Moudgil

**Affiliations:** 1 Department of Microbiology and Immunology, Department of Medicine, University of Maryland School of Medicine, Baltimore, MD 21201, USA; 2 Division of Rheumatology, Department of Medicine, University of Maryland School of Medicine, Baltimore, MD 21201, USA

## Abstract

Complementary and alternative medicine products are increasingly being used for the treatment of autoimmune diseases. However, the mechanisms of action of these agents are not fully defined. Using the rat adjuvant arthritis (AA) model of human rheumatoid arthritis, we determined whether the ethanol extract of *Celastrus aculeatus *Merr. (Celastrus), a Chinese herb, can down-modulate the severity of AA, and also examined the Celastrus-induced changes in immune responses to the disease-related antigen mycobacterial heat-shock protein 65 (Bhsp65). AA was induced in the Lewis (LEW; RT.1^l^) rat by immunization subcutaneously with heat-killed *M. tuberculosis *H37Ra (Mtb). Celastrus was fed to LEW rats by gavage daily, beginning either before Mtb challenge (preventive regimen) or after the onset of AA (therapeutic regimen). An additional group of rats was given methotrexate for comparison. All rats were graded regularly for the signs of arthritis. In parallel, the draining lymph node cells of Celastrus-treated rats were tested for proliferative and cytokine responses, whereas their sera were tested for the inflammatory mediator nitric oxide. Celastrus feeding suppressed both the induction as well as the progression of AA, and the latter effect was comparable to that of methotrexate. Celastrus treatment induced relative deviation of the cytokine response to anti-inflammatory type and enhanced the production of anti-Bhsp65 antibodies, which are known to be protective against AA. Celastrus feeding also reduced the levels of nitric oxide. On the basis of our results, we suggest further systematic exploration of Celastrus as an adjunct therapeutic modality for rheumatoid arthritis.

## Introduction

Rheumatoid arthritis (RA) is a chronic debilitating autoimmune disorder that affects about 2.1 million Americans [1-5]. The drugs commonly in use for the treatment of RA include glucocorticoids (for example, cortisone and prednisone), non-steroidal anti-inflammatory drugs (NSAIDS; for example, ibuprofen and naproxen), disease-modifying anti-rheumatic drugs (DMARDs; for example, methotrexate (MTX) and leflunomide), and biological response modifiers (for example, tumor necrosis factor-αblocking agents) [6,7]. However, besides their high cost, the prolonged use of many of these drugs is associated with severe adverse reactions and toxicity, including some risk of infections in subsets of patients being treated with biological response modifiers [6,7]. As a result, alternative treatments based on natural plant products and herbal mixtures belonging to the realm of complementary and alternative medicine (CAM) are becoming increasingly popular in the US and other countries [2-5,8]. However, there is skepticism about CAM products in the minds of both the public as well as the scientific community, mostly because the mechanisms of action of many of these products are poorly defined, or not at all. Thus, there is a need to systematically study and define the mechanisms underlying the activity of CAM products that have been used for the treatment of rheumatic diseases in folk medicine around the world for centuries.

*Celastrus aculeatus *Merr. (Celastrus) [9-15] is a Chinese medicine that belongs to the family Celastraceae and the genus *Celastrus*. The roots, stem, and leaves of Celastrus have been used in folk remedies in China for centuries to treat RA, osteoarthritis, lower back pain, and so on. Celastrus and some of its defined constituents possess anti-inflammatory, anti-oxidant, and anti-cancer properties [9-15]. However, the mechanisms underlying the anti-arthritic activity of Celastrus have not been fully examined. Considering that RA is an autoimmune disease resulting from a dysregulated immune system [6,7,16,17], it is imperative to examine the immunological basis of Celastrus or any other new potential anti-arthritic therapeutic agent under consideration.

Animal models of RA have contributed significantly both to our understanding of the pathogenesis of autoimmune arthritis as well as to the testing of new therapeutic agents of natural or synthetic origin [18-21]. As a pre-requisite to unraveling the mechanisms underlying the beneficial effects of Celastrus in RA, we set out to first validate the anti-arthritic activity of Celastrus under controlled experimental conditions using a well established model of RA, adjuvant-induced arthritis (AA), which can be induced in the Lewis (LEW; RT.1^l^) rat by subcutaneous (s.c.) immunization with heat-killed *M. tuberculosis *H37Ra (Mtb) [18,22-26]. Thereafter, we examined in LEW rats with AA the effects of Celastrus on the T cell and antibody responses to the disease-related antigen mycobacterial heat-shock protein 65 (Bhsp65) [18,22-25], which also is the target of T cell and antibody response in RA patients [27,28].

Our results show that Celastrus can induce protection against arthritis both in the preventive as well as in the therapeutic setting, and that this beneficial anti-arthritic effect of Celastrus is attributable in part to modulation both of the immune response to the disease-related antigen Bhsp65 [22-25] and one of the mediators of inflammation and tissue damage, nitric oxide (NO) [29,30].

## Materials and methods

### Rats

Inbred male Lewis (LEW/SsNHsd; LEW; RT.1^1^) rats (5 to 6 weeks old, 130 to 160 g) were procured from Harlan Sprague Dawley (Indianapolis, IN, USA), and then maintained in the vivarium facility of the University of Maryland School of Medicine (UMB). All procedures performed on these animals were in accordance with the guidelines of the institutional animal care and use committee (IACUC).

### Adjuvant/antigen

Mtb was obtained from Difco Laboratories (Detroit, MI, USA). Bhsp65 was prepared from BL21 (DE3) pLysS cells (Novagen, Madison, WI, USA) transformed by the vector pET23b-GroEL2 (Colorado State University, Fort Collins, CO, USA) [31]. Synthetic peptide 177–191 of Bhsp65 (B177) and other Bhsp65 peptides were obtained from Global Peptide Services (Fort Collins, CO, USA). Purified protein derivative was purchased from Mycos Research (Fort Collins, CO, USA), whereas hen egg white lysozyme (HEL) and keyhole limpet hemocyanin (KLH) were obtained from Sigma-Aldrich (St. Louis, MO, USA).

### Induction and evaluation of adjuvant arthritis

LEW rats were immunized s.c. at the base of the tail with 200 μl (1 mg/rat) of Mtb in mineral oil, and then observed regularly for clinical signs of arthritis like erythema, swelling and induration [24,32]. The severity of arthritis in each paw was graded on a scale from 0 to 4. The maximum arthritic score for each paw was 4, and the total arthritis score per rat was 16.

For histological assessment of arthritis, hind paws of rats were harvested, fixed for 3 days in a solution containing 10% formalin, HCl, and H_2_O (10:2:88, v/v), and then embedded in paraffin. Serial paraffin sections (7 μm; Leica RM2135, Leica Instruments, Germany) were stained with hematoxylin and eosin, and then examined and graded under the microscope for histopathological changes in the joints [18], including inflammatory cell infiltrate, synovial hyperplasia, cartilage damage and bone erosion [33,34]. Each of these parameters was graded on a scale from 0 to 3 as follows: 0 = absent; 1 = mild; 2 = moderate; and 3 = severe [33,34]. For each rat, a histological section of either the left or the right hind paw was examined and the results are presented as median (interquartile range).

### Preparation and characterization of the ethanol extract of Celastrus

The roots and stems of *Celastrus aculeatus *Merr. were collected in the Guangdong province of China, and their identity was confirmed by Dr Ye Hua-gu, a plant taxonomist at South China Institute of Botany, the Chinese Academy of Sciences, Guangzhou. The dried roots and stems were minced with a grinder and then the powder was extracted for 2 h with 75% ethanol. The ethanol extract was collected, and the procedure was repeated twice. The final ethanol extract was condensed with a rotary evaporator, and the concentrated extract was dried. The presence of three of the major groups of components of Celastrus, namely triterpenes (for example, celastrol, celasdin C), flavonoids (for example, epiafzelechin), and sesquiterpenes (for example, orbiculin F) [9-15] was confirmed by HPLC and LC/MS analysis (data not shown). However, to assess the anti-arthritic activity of the natural mixture of the constituents in the ethanol extract, rats were fed with unfractionated crude extract. For this reason, the amount of Celastrus extract fed per rat was relatively high. The LD50 for the Celastrus extract was found to be 55.7 g/kg. After performing pilot experiments on the modulation of AA with different doses of Celastrus ranging from 0.5 to 3 g/kg, the two doses finally selected for use in this study corresponded to the LD50 dose as follows: 1.5 g/kg (1/37 of LD50) and 3 g/kg (1/18.5 of LD50).

### Feeding of Celastrus to LEW rats

#### Prevention regimen

Naïve LEW rats were fed Celastrus (experimental group; 1.5 or 3 g/kg body weight) or the vehicle (water; control group) using a gavage needle (FNC-16-3, Kant Scientific Corporation, Torrington, CT, USA) once daily for 4 days prior to s.c. immunization with Mtb and then continued uninterrupted for the entire duration of the observation period. Following Mtb challenge, all rats were graded regularly for clinical signs of arthritis [24,32].

#### Therapeutic regimen

Naïve LEW rats were challenged with Mtb s.c. for the induction of AA. Beginning at the onset of AA, and then continued throughout the course of AA, the experimental group of rats was fed Celastrus (1.5 or 3 g/kg) daily by gavage, whereas the control group received the vehicle (water). A third group of arthritic rats was fed MTX (0.5 mg/kg), an established anti-arthritic compound, as a positive control. All these rats were observed regularly for signs of arthritis throughout the period of feeding with Celastrus/water.

### Lymph node cell proliferation assay

LEW rats were immunized s.c. with Mtb (1 mg/rat). The draining lymph nodes (inguinal, para-aortic, and popliteal) of sub-groups of these rats were harvested on day 8, 12 or 24 after injection, and a single cell suspension of lymph node cells (LNCs) was prepared [32]. These LNCs (2.5 × 10^5 ^to 5 × 10^5 ^cells/well) were tested in a proliferation assay in HL-1 serum-free medium (BioWhittaker, Walkersville, MD, USA) in the presence or absence of antigen [32]. Purified protein derivative was used as a positive control, whereas HEL served as a negative control. The results were expressed either as counts per minute (cpm) or as a stimulation index (the ratio of cpm in the presence of antigen and cpm of cells in medium alone).

### Measurement of cytokine levels by ELISA

LNCs of Celastrus-treated or control rats were plated in a 96-well plate as for the LNC proliferation assay described above. After 72 h of culture of cells with specific antigens, the culture supernatant was collected and tested for IFN-γ and IL-10 using ELISA kits (Biosource International, Camarillo, CA, USA) following the manufacturer's instructions [32]. After the last reaction, the color intensity (optical density) was measured at 450 nm with an automated Coulter ELISA Reader (Coulter Electronics, Kendall, FL, USA) and the results were expressed as pg/ml.

### ELISA for anti-Bhsp65 antibodies

A flat-bottom 96-well microtiter ELISA plate was coated with 100 ng/well each of purified Bhsp65 (test antigen) or KLH (control antigen) in phosphate-buffered saline (pH 7.2) for 16 h at 4°C [31]. After washing, the wells were blocked for 3 h at room temperature with 10% bovine serum albumin (EIA grade; Sigma-Aldrich) in phosphate-buffered saline. The sera were tested at dilutions ranging from 1:50 to 1:8,100. The plate-bound antibody was detected using goat anti-rat immunoglobulin conjugated to horseradish peroxidase (BD Pharmingen, San Diego, CA, USA). Thereafter, the substrate was added for color development, and after 15 minutes the reaction was stopped with 0.5 M sulfuric acid. The color intensity (optical density) was read at 540 nm using an ELISA reader.

### Determination of NO levels in serum and LNC culture supernatant

A cohort of LEW rats was fed Celastrus or water following the above-mentioned 'prevention' regimen and then two types of samples were collected, as follows: for serum, these rats were bled at days 8, 16 and 24 after injection with Mtb and then sera were separated from the clotted blood; and for culture supernatant, the draining LNCs harvested from sub-groups of these rats on day 8, 16 or 24 after Mtb injection were restimulated *in vitro *for 72 h with Bhsp65 (test antigen) or HEL (control antigen), and the culture supernatant was collected. The levels of NO in these samples were then evaluated by measuring the nitrite (NO_2_^-^) and nitrate (NO_3_^-^) content by using a colorimetric assay kit (Biovision research products, Mountain View, CA, USA). The results were expressed as μM.

### Statistical analysis

Student *t*-test and Wilcoxon rank sum test were used to analyze the data obtained from different experiments. The results were considered significant at *p *< 0.05.

## Results

### Celastrus suppresses the induction of AA in the LEW rat

**Figure 1:**
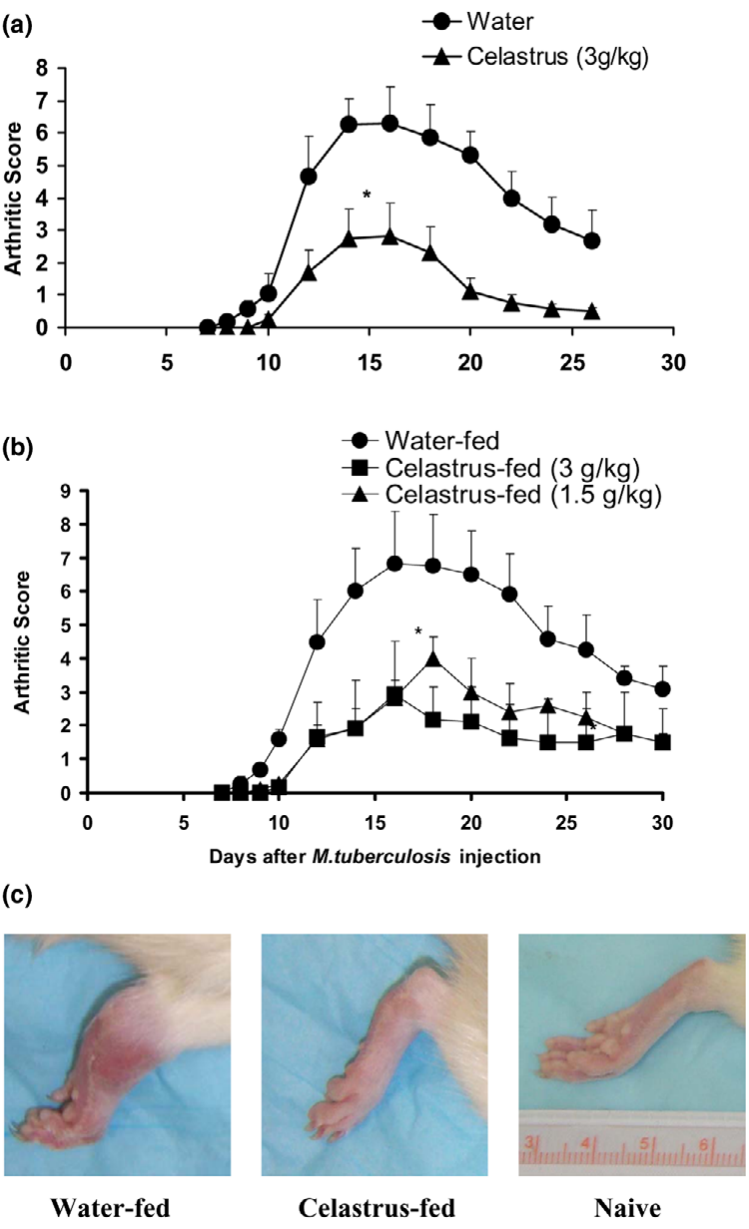
Feeding of Celastrus suppresses the induction of adjuvant arthritis (AA) in the Lewis (LEW) rat. **(a) **LEW rats (*n *= 7 per group) were fed by gavage daily either Celastrus (triangles; 3 g/kg body weight, experimental group) or water (circles; control group) starting on day 4 prior to *M. tuberculosis *H37Ra (Mtb) immunization (1 mg/rat) and then continuing throughout the observation period. Following Mtb injection, these rats were scored regularly for signs of arthritis. The difference in the mean arthritic scores of the Celastrus-fed and Water-fed rats during the course of AA was significant (*p < 0.05 by Wilcoxon rank sum test). **(b) **The results of an independent repeat experiment including two groups of Celastrus-treated rats are shown in this section. The difference in arthritic scores of Celastrus-fed versus Water-fed rats was significant (*p < 0.05) for each of the groups tested (triangles; 1.5 g/kg, *n *= 4; squares, 3 g/kg, *n *= 4). **(c) **The photograph shows the hind paw of a representative LEW rat from the water-fed (left) and Celastrus-fed (middle) groups on day 16 after Mtb immunization. The hind paw of a naive LEW rat (right) is also shown for comparison; each unit on the scale equals 1 mm, with 10 units between numbered marks.

To examine the effect of Celastrus on the initiation and progression of AA, naïve LEW rats were fed daily either Celastrus (1.5 or 3 g/kg body weight per day) or the vehicle (water; control group) starting day 4 prior to Mtb immunization, and then continued throughout the course of the disease. In the period following Mtb injection, all rats were observed regularly for signs of arthritis. Celastrus-fed rats showed significantly reduced disease severity compared to that of water-fed control rats (Figure 1a–c). The effect of Celastrus on clinical arthritis was also validated by histological examination of arthritic joints. The results (Table 1) show that synovial infiltration by mononuclear cells and the damage to cartilage and bone were significantly reduced in Celastrus-treated rats compared to that in control water-fed rats. Thus, feeding Celastrus to LEW rats significantly reduced the severity of subsequently induced AA.

**Table 1:** Quantification of histological changes in the hind paws of Celastrus-fed (experimental) versus water-fed (control) Lewis rats

Group (n)	Cellular infiltrate^a^	Synovial hyperplasia^a^	Cartilage damage^a^	Bone erosion^a^
Water-fed				
Day 12 (10)	2 (1–3)	3 (2–3)	2.5 (0–3)	2.5 (0–3)
Day 24 (10)	3 (2–3)	3 (3–3)	3 (1–3)	3 (0–3)
				
Celastrus-fed				
Day 12 (10)	1 (0–2)*	0 (0–2)*	0 (0–2)*	0 (0–1)*
Day 24 (9)	1 (0–3)*	1 (0–2)*	1 (0–2)*	1 (0–1)*

The results shown are the median (interquartile range) scores of histological sections of the left or the right hind paw using the grading system described in 'Materials and methods'. ^a^Histological grading of each parameter was done as follows: 0 = absent; 1 = mild; 2 = moderate; 3 = severe. *p < 0.01, comparing the results of the respective Celastrus-fed versus water-fed groups.

To define the mechanisms underlying the anti-arthritic activity of Celastrus, we examined the changes in the immune response to the disease-related antigen Bhsp65 as well as in the production of a mediator of inflammatory arthritis (NO) in Celastrus-treated LEW rats. The results of these investigations are described below.

### Celastrus feeding to LEW rats induces preferential secretion of anti-inflammatory cytokines over pro-inflammatory cytokines in response to Bhsp65

To test and compare the T cell proliferative and cytokine response to the disease-related antigen Bhsp65 of Celastrus-treated versus control (water-fed) LEW rats, the draining LNCs of these arthritic rats were tested using the appropriate assays on day 12 after Mtb immunization. The two groups of rats had comparable (*p *> 0.05) levels of proliferative response to Bhsp65/B177–191 (data not shown).

**Figure 2:**
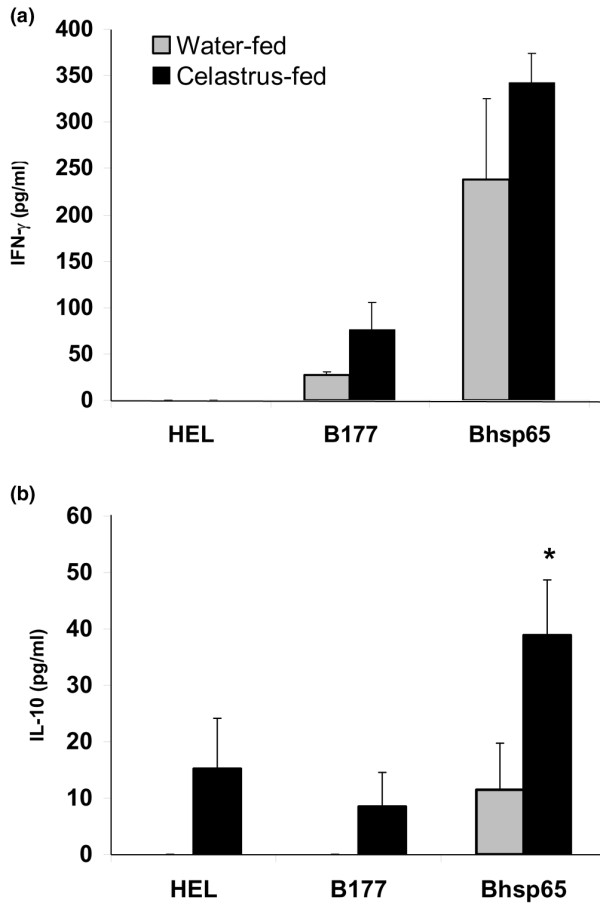
The cytokine response to mycobacterial hsp65 (Bhsp65) of lymph node cells (LNCs) of Celastrus-fed versus water-fed Lewis (LEW) rats. Two groups of LEW rats (*n *= 6 to 9) were fed either Celastrus (3 g/kg) or water as described in the legend to Figure 1. A sub-group of these LEW rats was euthanized on day 12 after *M. tuberculosis *H37Ra immunization, and the draining LNCs of these rats were cultured in a 96-well plate in the presence of the indicated recall antigens (HEL, hen eggwhite lysozyme; B177, synthetic peptide 177–191 of Bhsp65). The supernatant was collected after 72 h of cell culture and tested in ELISA for **(a) **IFN-γ and **(b) **IL-10. The results are expressed as pg/ml (mean + standard error of the mean). The difference in the level of IL-10 but not of IFN-γ in response to Bhsp65 in Celastrus-fed versus water-fed rats is statistically significant (*p < 0.05). The mean IFN-γ/IL-10 ratio in response to Bhsp65 of Celastrus-treated (8.79) rats was significantly reduced compared to that of the water-fed (20.76) rats.

In regard to the cytokine response to Bhsp65, the levels of IFN-γ (a pro-inflammatory cytokine) of the Celastrus-fed and water-fed rats were comparable (Figure 2a), but the levels of IL-10 (an anti-inflammatory cytokine; Figure 2b) were significantly (*p *< 0.05) up-regulated in Celastrus-fed rats compared to water-fed rats. The mean ratio of IFN-γ and IL-10 secreted in recall response to Bhsp65 by Celastrus-fed rats (ratio 8.79) was significantly lower than that of the water-fed rats (ratio 20.76), demonstrating that Celastrus preferentially facilitated the secretion of IL-10 and, thereby, induced a relative skewing (immune deviation) of the cytokine response towards a predominantly anti-inflammatory type.

### Celastrus-fed LEW rats reveal enhanced antibody response to Bhsp65

**Figure 3:**
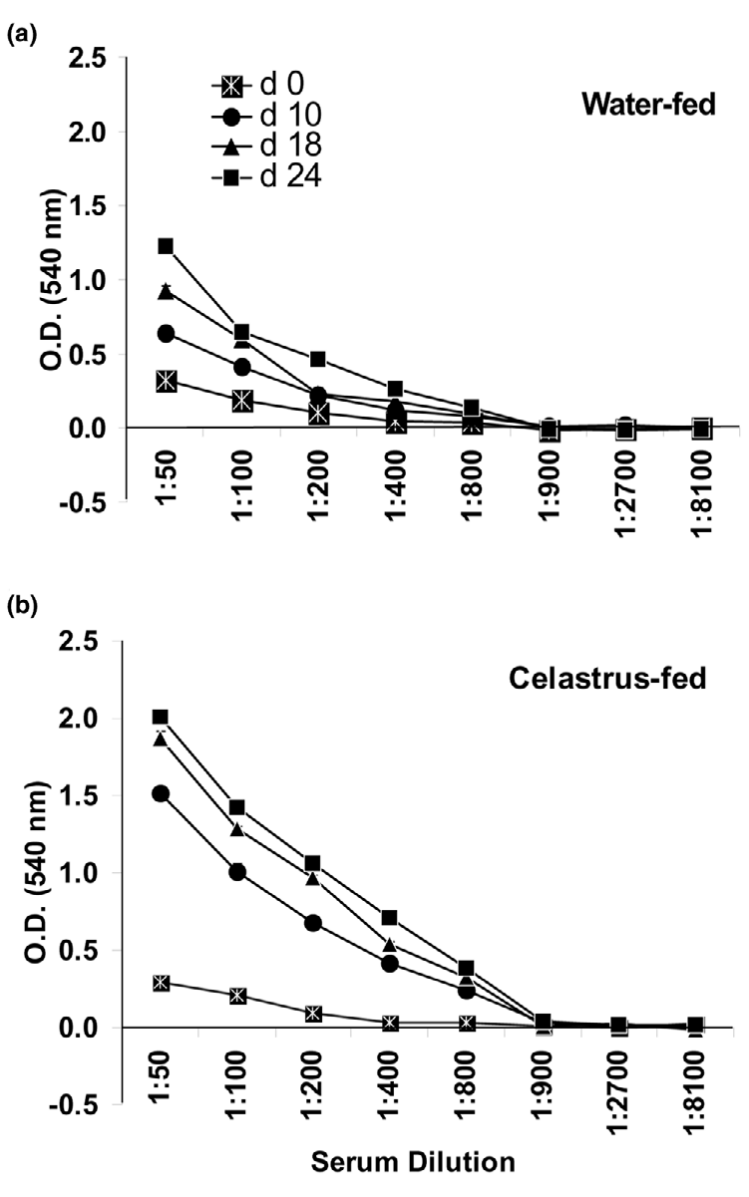
Antibody response to mycobacterial hsp65 (Bhsp65) of Celastrus-fed Lewis (LEW) rats. LEW rats (*n *= 4 to 6) were fed either Celastrus (3 g/kg) or water as described in the legend to Figure 1. Blood samples were collected from LEW rats immediately before (preimmune serum; day 0) challenge with *M. tuberculosis *H37Ra (Mtb; 1 mg/rat) as well as at different time points thereafter (days 10, 18 and 24). These sera were tested separately at different dilutions (1:50 to 1:8,100) by ELISA for total immunoglobulin against Bhsp65. The results are expressed as optical density (O.D.) at 540 nm (mean + standard error of the mean). At one representative concentration of sera (for example, 1:100 dilution), the level of antibody response to Bhsp65 in Celastrus-fed rats was significantly higher than that of water-fed rats on days 18 and 24 (**p < 0.01 each).

LEW rats were immunized s.c. with Mtb (1 mg/rat) after 4 days of daily feeding of either Celastrus or water. Thereafter, these rats continued to receive daily either Celastrus or water. The sera collected from these rats at specific time points before and after Mtb immunization were tested at different dilutions, ranging from 1:50 to 1:8,100, by ELISA for total immunoglobulin against Bhsp65. The results (Figure 3) show that the levels of anti-Bhsp65 antibodies increased gradually from day 0 through day 24. At both 1:100 and 1:200 serum dilutions, the level of antibody response to Bhsp65 in Celastrus-treated rats was significantly (*p *< 0.01) higher than that of water-fed rats on day 18 and day 24. These anti-Bhsp65 antibodies were composed mostly of IgG (data not shown). However, as expected, the sera from both the test and control group of rats had only minimal reactivity against KLH (control antigen), with no significant difference between the two groups of rats (data not shown). Thus, the increased antibody response to Bhsp65 was associated with the Celastrus-induced protection against AA.

### Reduced levels of NO in serum and LNC culture supernatant of Celastrus-treated LEW rats

**Figure 4:**
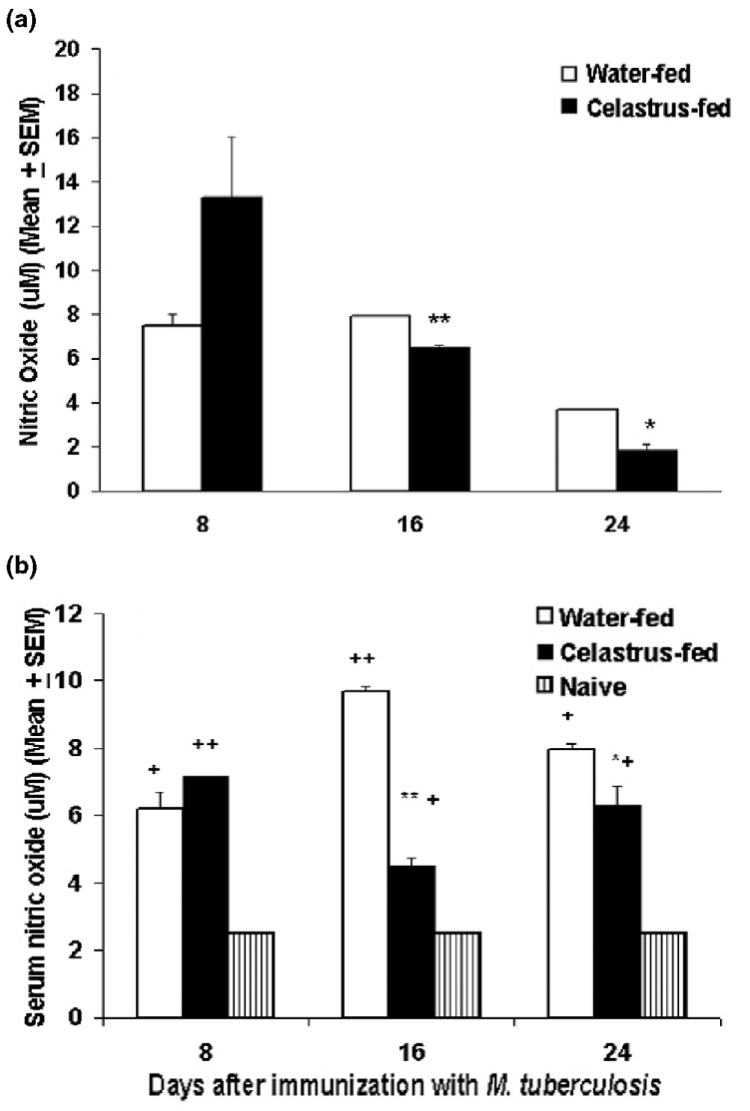
Levels of nitric oxide (NO) in lymph node cell (LNC) culture supernatant and serum of Celastrus-treated Lewis (LEW) rats. **(a) **LNC culture supernatant and **(b) **sera were obtained from Celastrus-fed and Water-fed rats as described in Materials and methods. The level of NO in these samples was determined by a colorimetric assay. The results are presented as μM (mean + standard error of the mean). The level of NO secreted into the culture supernate following mycobacterial hsp65 (Bhsp65) restimulation of LNCs of Celastrus-fed rats was significantly (**p < 0.005, *p < 0.05) lower than that of water-fed rats on days 16 and 24 following *M. tuberculosis *H37Ra (Mtb) immunization (a). The levels of NO in sera of Celastrus-fed rats was significantly (**p < 0.01, *p < 0.05) decreased at days 16 and 24 compared to those of water-fed rats (b). However, the levels of NO in sera of both these Mtb-immunized groups of rats were higher (++*p *< 0.01, +*p *< 0.05) compared to those of naïve sera. In each section, some of the error bars are too small to be detected.

NO production is increased in patients with RA, and its production correlates with the severity of arthritis [29,30,35]. Therefore, we reasoned that Celastrus might down-modulate AA, in part by inhibiting the production of NO, and tested this proposition in Celastrus-fed LEW rats. Our results show that the levels of NO in the culture supernatant of LNCs of Mtb-primed rats restimulated *in vitro *with Bhsp65 were significantly lower in Celastrus-fed rats than that in water-fed rats on days 16 and 24 (Figure 4a). Similarly, the level of serum NO in Celastrus-fed rats was significantly lower than that in water-fed rats on days 16 and 24 (Figure 4b). However, the NO levels in the sera of both these groups were much higher than that in sera of naïve rats (Figure 4b). Taken together, these results show that arthritic LEW rats produced NO in response to Bhsp65, and that Celastrus feeding reduced the levels of NO. This decrease in NO levels in turn correlates with the reduced severity of arthritis in Celastrus-treated rats compared to control rats.

### Celastrus feeding suppresses the severity of ongoing AA in the LEW rat and the level of this effect is comparable to that of MTX

**Figure 5:**
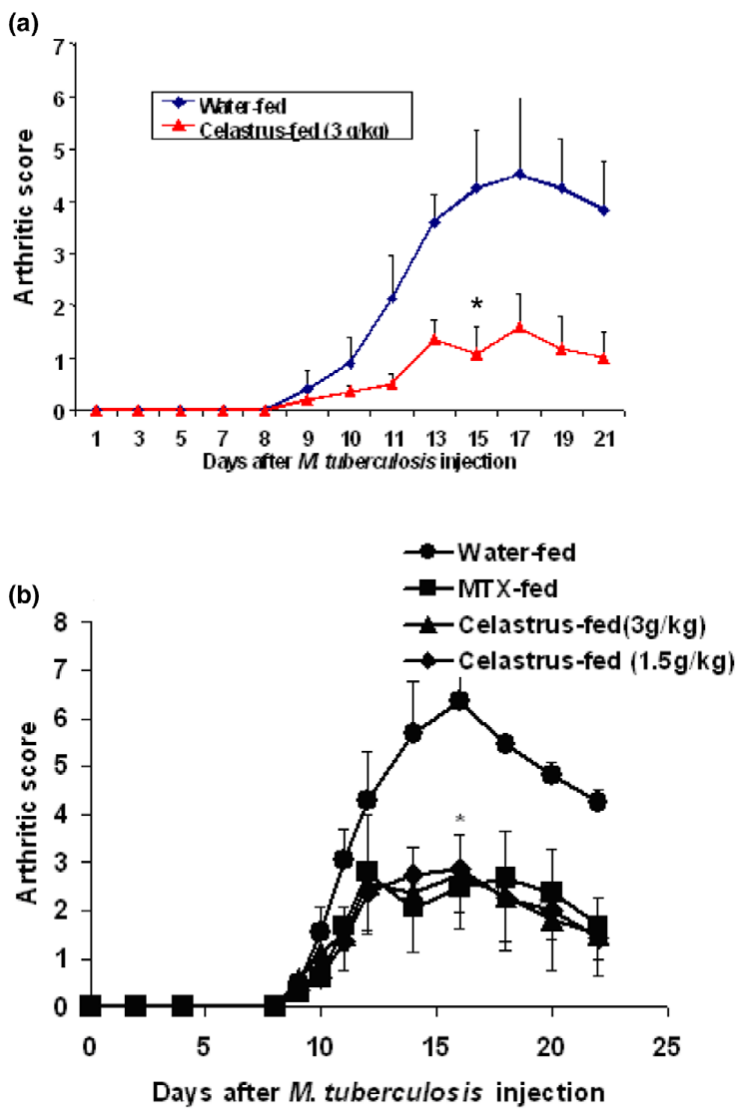
Celastrus induces therapeutic down-modulation of adjuvant arthritis (AA) that is comparable to that when using methotrexate (MTX). A cohort of Lewis (LEW) rats was immunized subcutaneously with *M. tuberculosis *H37Ra (Mtb; 1 mg/rat) at the base of the tail and then split into different groups (*n *= 4 per group). Beginning day 9 thereafter, coinciding with the onset of clinical signs of arthritis in the hind paws, these rats were fed daily by gavage either Celastrus (experimental group) or water (negative control group). **(a) **Experimental rats were fed with 3 g/kg of Celastrus, or **(b) **with either 3 or 1.5 g/kg of Celastrus. An additional group of experimental rats shown in (b) received MTX (0.5 mg/kg; positive control). All these rats were observed and scored regularly for the severity of AA. In both (a) and (b) the difference in the mean arthritic score of each of the Celastrus-fed versus water-fed group of rats was significant (*p < 0.05 by Wilcoxon rank sum test). Similarly, in (b), a significant (*p < 0.05) difference in arthritic scores was observed between MTX-fed and water-fed rats, whereas comparable (*p *> 0.05) arthritic scores were observed for Celastrus-fed versus MTX-fed rats.

We have described above that feeding of Celastrus to naïve LEW rats beginning prior to the induction of AA by Mtb injection can afford protection against AA (Figure 1). However, from the clinical viewpoint of RA patients, it is critical that a potentially beneficial anti-arthritic product displays not only a preventive effect but also a therapeutic effect by suppressing ongoing (established) arthritis. In this regard, we examined the therapeutic potential of Celastrus in the AA model. Naïve LEW rats were challenged with Mtb s.c. for the induction of AA. Beginning at the onset of AA, and then continuing throughout its course, one of the experimental groups of rats was fed Celastrus (test group) and the control group received the vehicle (water). Another group of experimental rats was fed an established anti-arthritic compound, MTX (positive control). All these rats were observed regularly for signs of arthritis. The results (Figure 5a,b) show that both the Celastrus-fed and the MTX-fed experimental groups had a significantly decreased severity of AA compared to the Water-fed control rats, and both the high (3 g/kg) and the low (1.5 g/kg) doses of Celastrus had comparable beneficial effects against AA (Figure 5b). The severity of the disease in each of these two experimental groups of rats (Celastrus-fed and MTX-fed) was significantly reduced compared to control (water-fed) rats (Figure 5b). Intriguingly, the level of the suppressive effect on arthritis of Celastrus was comparable to that of MTX. Thus, Celastrus showed both preventive as well as therapeutic anti-arthritic activity in the AA model.

## Discussion

Our results show that *Celastrus aculeatus *Merr. (Celastrus) suppresses the induction of AA when fed to LEW rats prior to Mtb challenge, as well as down-modulates the progression of AA when administered to arthritic rats at the onset of the disease. The significant reduction in the severity of clinical AA following Celastrus feeding was further validated by limited histological changes in the joints. Furthermore, the level of suppression of ongoing AA by Celastrus was comparable to that of MTX, a standard anti-arthritic agent used for the treatment of arthritis. This attribute of Celastrus is an important one because many regimens based on synthetic or natural compounds can successfully prevent the induction of arthritis, but they often fail to control the course of the ongoing disease. In this regard, Celastrus is a promising anti-arthritic agent that could be further explored as a therapeutic modality in controlled pilot clinical trials on RA patients. As this is our first study on the effect of Celastrus on AA, we have used the unfractionated ethanol extract of the roots and stems to preserve as much of the natural proportion of different constituents in the mixture as possible. Accordingly, the dose of Celastrus fed to rats is apparently high. However, in subsequent follow up studies, we plan to use one or more of the purified components of the crude extract. It has been reported by others that various components of Celastrus possess anti-inflammatory and anti-tumor properties, and these include a variety of sesquiterpene esters (for example, celastrol, celaphanol, celasdin, orbiculin, esters with the β-dihydroagarofuran skeleton) and flavonoids (for example, epiafzelechin) [9-15]. Some of the reported pathways inhibited by these components are mediated by nuclear factor kappa-B (NF-κB), inducible nitric oxide synthase (iNOS), and cyclooxygenase (COX) [12-15,35].

We observed that the suppression of clinical arthritis in Celastrus-fed LEW rats was associated with significant changes in the immune response to Bhsp65. Furthermore, both the cell-mediated and the antibody responses to Bhsp65 were affected. AA is driven by pro-inflammatory cytokines (IFN-γ and tumor necrosis factor-α); in this context, Celastrus treatment facilitated the secretion of the anti-inflammatory cytokine IL-10 over the pro-inflammatory cytokine IFN-γ, resulting in the overall skewing (immune deviation) of the cytokine response to an anti-inflammatory type [26]. This relative deviation of the cytokine response, caused either by decreased Th1-type cytokines and/or by enhanced Th2-type cytokines leading to the regression of an autoimmune disease, is reminiscent of other compounds of synthetic (for example, peptides of antigenic proteins or cytokines) [25,36,37] or natural origin [38,39] that can successfully control disease in animal models of arthritis.

Celastrus feeding to LEW rats immunized with Mtb led to enhanced production of antibodies to Bhsp65 compared to the control water-fed rats. Thus, a decrease in inflammatory arthritis in LEW rats was associated with an increase in the anti-Bhsp65 antibody response. This inverse association is supported by previous work by others [25] and us [31] demonstrating that anti-Bhsp65 antibodies produced during the course of AA are disease-protective rather than being pathogenic in nature. Unlike in other animal models of RA in which antibodies are arthritogenic [40,41], in the AA model certain subsets of anti-Bhsp65 antibodies generated either during the course of AA [25,31] or following AA-protective tolerization with Bhsp65 [42] contribute to disease regulation. It has been proposed that the protective effect of antibodies in AA is probably mediated by the induction of IL-10 production from mononuclear cells [25]. In this regard, our finding of a Celastrus-induced deviation of the cytokine response of arthritic LEW rats towards IL-10 correlates very well with our observation of enhanced anti-Bhsp65 antibody response in Celastrus-treated rats, and the observed immune deviation towards IL-10 might be attributable, in part, to the increased antibody response to Bhsp65. We further suggest that the anti-Bhsp65 antibodies might also contribute to the protection against AA by modulating antigen processing and presentation [43] and, thereby, facilitating the induction of the immune response to one or more of the regulatory T cell determinants within Bhsp65 previously identified by others [23,25,36] and us [24]. Thus, changes in both the cell-mediated and the antibody responses to Bhsp65 following Celastrus feeding might cooperate to down-regulate the severity of AA in the LEW rat.

In addition to the disease-regulating changes in the immune response to Bhsp65, the beneficial effect of Celastrus in AA was also related to inhibition of the production of a well known mediator of inflammation, namely NO [20,29,30,35]. We observed antigen specificity in the production of NO; Bhsp65-restimulated LNCs of Mtb-immunized water-fed (control) LEW rats produced significantly higher levels of NO than those restimulated by the control antigen, HEL. Furthermore, Celastrus treatment significantly reduced the levels of NO in both LNC culture supernatant and sera of Mtb-immunized rats. Taken together, these results document not only a direct association between the levels of NO and the severity of AA, but also provide insight into the *in vivo *anti-inflammatory activity of Celastrus. These results of Celastrus-mediated suppression of NO production *in vivo *are further corroborated by reports by other investigators showing a similar effect of Celastrus *in vitro *using macrophage cell lines (for example, RAW cells) [12,15]. Furthermore, it has been reported that oral feeding of B6 mice with the ethyl acetate extract of *Tripterygium wilfordii *Hook F (TWHF) or its active component, triptolide, led to the inhibition of both NO production and iNOS mRNA expression by macrophages [44], and this decrease in NO production was implicated in mediating the anti-inflammatory effects of TWHF. One of the mechanisms by which Celastrus leads to decreased NO production might involve NF-κB, which controls the expression of genes encoding inducible enzymes, such as iNOS and COX, which in turn generate some of the critical mediators of the inflammatory response [14,15,35]. In fact, some of the active components of Celastrus have been shown to serve as inhibitors of the NF-κB pathway (for example, celastrol and celaphanol A) [12,15] and the COX pathway (for example, epiafzelechin) [13]. In addition, NF-κB activity is inversely related to that of the heat-shock response as the induction of heat-shock proteins is associated with a decrease in NF-κB activity [14]. Celastrol can lead to the induction of heat-shock protein gene expression by activation of heat-shock factor-1 [14], and the enhanced response to self hsp65 can, in turn, contribute to protection against AA [23,32]. Thus, by regulating the activity of NF-κB, Celastrus apparently influences multiple inter-connected pathways that participate in the regulation of autoimmune arthritis.

Our results suggest that the ethanol extract of Celastrus as well as its individual components should be explored further for the treatment of RA through double-blind, placebo-controlled preclinical and clinical trials in RA patients following the strategy employed successfully by other investigators for translational research on TWHF [8,45]. There is a compelling need to fully examine multiple natural products such as TWHF and Celastrus for their potential as anti-arthritic agents because all RA patients may not respond equally well to any single herbal medicine owing to differences in body constitution and genetics, and each natural plant product may have unique compatibility with the standard mainstream medications when taken together. The availability of several different natural plant products having anti-arthritic activity would enlarge the scope of the use of CAM modalities for the treatment of RA in conjunction with conventionally used drugs.

## Conclusion

The ethanol extract of *Celastrus aculeatus *Merr. (Celastrus) has potent anti-arthritic activity. Feeding Celastrus to LEW rats offered protection against the subsequent induction as well as progression of AA. The therapeutic effect of Celastrus was comparable to that of MTX. Celastrus-induced protection against AA involved significant modulation of both the cytokine and antibody responses to the disease-related antigen Bhsp65. In addition, Celastrus suppressed the production of a known mediator of inflammation, NO. Celastrus should be further tested in clinical trials on patients with RA to explore its utility as a natural CAM product that might be beneficial either alone or in combination with conventionally used drugs, with the objective of complementing the beneficial anti-arthritic effects and reducing the side effects of the latter group of drugs.

## Abbreviations

AA = adjuvant arthritis; Bhsp65 = mycobacterial hsp65; CAM = complementary and alternative medicine; COX = cyclooxygenase; ELISA = enzyme-linked immunosorbent assay; HEL = hen eggwhite lysozyme; hsp65 = heat-shock protein 65; IFN = interferon; IL = interleukin; iNOS = inducible nitric oxide synthase; KLH = keyhole limpet hemocyanin; LEW = Lewis; LNC = lymph node cell; Mtb = *M. tuberculosis *H37Ra; MTX = methotrexate; NF-κB = nuclear factor kappa-B; NO = nitric oxide; RA = rheumatoid arthritis; s.c. = subcutaneous; TWHF = *Tripterygium wilfordii *Hook F.

## Competing interests

The authors declare that they have no competing interests.

## Authors' contributions

LT conducted all the experiments, recorded and analyzed the raw data, prepared graphics, and participated in the interpretation of data as well as writing of the manuscript. KDM participated in the planning of experiments, data analysis, interpretation of results and writing of the manuscript.
